# Anti-seizure medication is not associated with an increased risk to develop cancer in epilepsy patients

**DOI:** 10.1007/s00415-020-10379-4

**Published:** 2021-01-23

**Authors:** Jenny Stritzelberger, Johannes D. Lang, Tamara M. Mueller, Caroline Reindl, Vivien Westermayer, Karel Kostev, Hajo M. Hamer

**Affiliations:** 1 Department of Neurology, Epilepsy Center, Friedrich-Alexander-Universität Erlangen-Nürnberg (FAU), Schwabachanlage 6, 91054 Erlangen, Germany; 2 IQVIA, Epidemiology, Frankfurt am Main, Main Airport Center, Unterschweinstiege 2-14, 60549 Frankfurt am Main, Germany

**Keywords:** Epilepsy, Antiseizure medication, Carcinogenicity, Carbamazepine

## Abstract

**Objective:**

Whether anti-seizure medication (ASM) increases the risk for cancer has been debated for decades. While for some ASM, a carcinoma-promoting effect has been suspected, carcinoma-protective effects have been shown for other ASM. However, the issue remains unresolved as data from preclinical and clinical studies have been inconsistent and contradictory.

**Methods:**

We collected anonymous patient data from practice neurologists throughout Germany between 2009 and 2018 using the IMS Disease Analyzer database (QuintilesIMS, Frankfurt, Germany). People with epilepsy (PWE) with an initial cancer diagnosis and antiepileptic therapy prior to the index date were 1:1 matched with a control group of PWE without cancer according to age, gender, index year, Charlson Comorbidity Index, and treating physician. For both groups, the risk to develop cancer under treatment with different ASMs was analyzed using three different models (ever use vs. never use (I), effect per one (II) and per five therapy years (III).

**Results:**

A total of 3152 PWE were included (each group, *n* = 1,576; age = 67.3 ± 14.0 years). The risk to develop cancer was not significantly elevated for any ASM. Carbamazepine was associated with a decreased cancer risk (OR Model I: 0.699, *p* < .0001, OR Model II: 0.952, *p* = .4878, OR Model III: 0.758, *p* < .0004).

**Significance:**

Our findings suggest that ASM use does not increase the risk of cancer in epilepsy patients.

**Supplementary Information:**

The online version of this article (10.1007/s00415-020-10379-4) contains supplementary material, which is available to authorized users.

## Introduction

The relationship between epilepsy and cancer has been the subject of considerable debate for a long time [1, 2]. Besides epilepsy itself as a risk factor for cancer in persons with epilepsy (PWE) due to diagnostic procedures or the lifestyle of PWE, especially the propensity of anti-seizure medication (ASM) to promote or protect against cancer has been discussed in several previous animal and epidemiological studies with contradictory results [3–5].

Phenobarbital (PB), for example, has been under suspicion to promote liver cancer in rodents [6, 7], but so far, epidemiologic data did not support this relationship in humans [8]. Phenytoin (PHT) was classified as possibly carcinogenetic by the International Agency for Research on Cancer due to induction of lymphoma and liver cell cancer in rats [9, 10], and a few case studies reported association between PHT and certain cancer types in humans. A retrospective study assessing long-term treatment with benzodiazepines found a slightly increased cancer risk for clonazepam [11]. High doses of gabapentin were associated with pancreatic tumors in a rat model [12], but clinical data did not sufficiently support a potential carcinogenic effect of gabapentin in humans [13]. Therefore, clinical evidence for carcinogenicity in humans is neither consistent nor sufficient [5].

On the other hand, ASM like valproate (VPA) and carbamazepine (CBZ) has been reported to exert anti-proliferative effects on certain cancer cell lines via histone deacetylase (HDAC) inhibitory activities [14, 15]. PHT and CBZ inhibited Prostate-Specific-Antigen secretion in vitro [16]. Another study found protective effects against bladder cancer for PB therapy in smokers [17]. However, it remained again unclear whether these experimental results translate to clinically relevant cancer-suppressive effects of these drugs [5, 14, 18–21].

Taken together, despite a great body of preclinical data and mechanistically plausible considerations, evidence for human carcinogenicity is inconsistent and sparse and the association between ASM and cancer risk remains still unclear.

Our aim was, therefore, to evaluate the safety of long-term use of various ASM in terms of potential carcinogenetic effects. We retrospectively investigated the association between ASM prescriptions and the incidence of various cancer types in a large German cohort.

## Materials and methods

### Database

This study was based on data from the Disease Analyzer database (IQVIA®), which compiles drug prescriptions, diagnoses, and basic medical and demographic data obtained directly and in anonymous data format from computer systems used in practices of general practitioners and specialists throughout Germany [22]. The database covers approximately 3% of all outpatient practices in Germany. Diagnoses (according to International Classification of Diseases, 10th revision [ICD-10]), prescriptions (according to Anatomical Therapeutic Chemical (ATC) Classification system), and the quality of reported data are monitored by IQVIA. In Germany, the sampling methods used to select physicians' practices are appropriate for obtaining a representative database of general and specialized practices [22].

### Study population

**Fig. 1:**
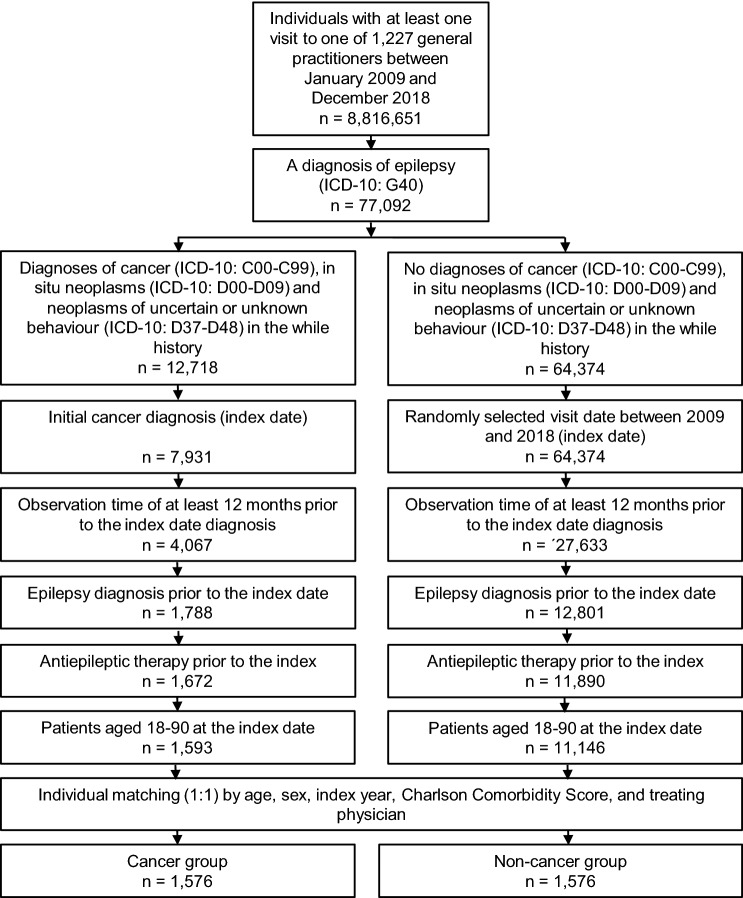
Flow chart of the study cohort, showing inclusion criteria and numbers of included epilepsy patients in both groups. ASM = antiseizure medication

This retrospective case–control study included patients with a diagnosis of epilepsy (ICD 10: G40) and a documented cancer diagnosis with the initial cancer diagnosis serving as the index date (ICD-10: C00-C97) from one of 1227 general practitioners between January 2009 and December 2018 (index date). Further inclusion criteria were as follows: (1) age 18–90 years at index date; (2) observation time of at least 12 months prior to the index date, and (3) epilepsy diagnosis and at least one ASM prescription prior to the index date (Fig. 1).

Using the same database, epilepsy patients without cancer were matched (1:1) to cancer cases by sex, age, index year, treating physician and a revised version of the Charlson Comorbidity Index (CCI) as a generic marker for comorbidity; the CCI describes 22 comorbid conditions where each condition is assigned a score from 1 to 6 depending on the risk of dying from it like congestive heart failure, diabetes, peripheral vascular disease or dementia [23]. The index date for the controls was a randomly selected visit date between 2009 and 2018 (Fig. 1).

### Study outcomes and covariates

The main outcome of the study was the association between the ASM use and cancer diagnosis. The prescriptions of all drugs licensed and marketed for the treatment of epilepsy in Germany in the study period were included. This comprised all drugs listed under the ATC-code N03 “Antiepileptics” when they were available in Germany during the study period. Thus, the analyses included carbamazepine, clonazepam, gabapentin, lacosamide, lamotrigine, levetiracetam, oxcarbazepine, phenobarbital, phenytoin, pregabalin, primidone, topiramate, and valproate.

### Statistical analyses

Descriptive analyses were performed for demographic and clinical variables, and the differences between cases and controls were evaluated using chi-squared tests for categorical variables and the Wilcoxon signed-rank test for age and CCI. Three regression models were used to analyze the association between ASM use and cancer risk. ASMs were included as a dichotomous variable in Model 1 (ever versus never use), as a continuous variable in Model 2 (therapy duration in years), and as dichotomous variable in Model 3 (at least 5 years of therapy vs. < 5 years of therapy). Regression models were performed separately for cancer in total as well as most frequent cancer sites including digestive organs, respiratory organs, skin, breast and prostate. In the regression analyses, odds ratios (OR) > 1.0 indicated a cancer-promoting effect while an OR < 1.0 implied a protective effect of the ASM observed. Results were regarded relevant when the significance level was < 0.01 and OR were < 0.85 or > 1.15, respectively. A p value of < 0.05 was considered statistically significant. All analyses were carried out using SAS 9.4 (SAS Institute, Cary, USA).

## Results

### Patient characteristics

From a total of 77,092 PWE in our database, 1576 PWE fulfilled the inclusion criteria (Fig. 1). The mean age was 67.3 years, with 70.1% being older than 60 years. 44.5% were females. The CCI was 3.7. 1576 PWE served as matched controls. The average duration of administration of ASM prior to the cancer diagnosis was 9.4 years (SD 5.8) with a maximal duration of 27.2 years.

### Anti-seizure medication

Levetiracetam (LEV) was the most frequently administered ASM in both groups (cancer group: 36.0%, non-cancer group: 32.2%; *p* < 0.027), followed by CBZ (24.6 vs. 30.9%; *p* < 0.001), VPA (21.5 vs. 21.0%; *p* < 0.760) and LTG (14.3 vs. 15.1%; *p* < 0.546). There were no significant differences regarding the administration of specific ASM in both groups except for CBZ which was prescribed more frequently in the non-cancer group (Table 1).

**Table 1:** Proportions of different antiseizure drugs prescribed in epilepsy patients (cancer patients and non-cancer controls)

Variable	Proportions in cancer patients (%)	Proportions in non-cancer patients (%)	P value
N	1573	1573	
Carbamazepine	24.6	30.9	< 0.001
Clonazepam	3.6	4.0	0.577
Gabapentin	10.9	11.2	0.820
Lacosamide	2.0	2.4	0.468
Lamotrigine	14.3	15.1	0.546
Levetiracetam	36.0	32.2	0.027
Oxcarbazepine	4.3	4.9	0.394
Phenobarbital	1.3	1.6	0.552
Phenytoin	6.2	8.2	0.033
Pregabalin	7.7	7.7	0.947
Primidone	4.8	4.5	0.673
Topiramate	2.7	3.3	0.348
Valproate	21.5	21.0	0.760

### Risk of anti-seizure medication for cancer

Exposure to ASM did not lead to a significant increase in total cancer incidence or in the organ-specific cancer subgroups in any of the three models (ever use vs never use, effect per 1 year of therapy, effect per 5 therapy years) with all OR being smaller than 1.1 (Table 2). The cancer risk did not differ in patients taking a combination of two or more ASM compared to those on monotherapy (OR 0.74, CI 0.49–1.11, *p* < 0.147).

**Table 2:** Association between antiseizure medication and the incidence of cancer in epilepsy patients in German general practices

ASM	Model I: ever use versus never use				Model II: Effect per year of therapy				Model III: Effect per 5 therapy years			
	OR	95% CI		*p* value	OR	95% CI		*p* value	OR	95% CI		*p* value
Carbamazepine	0.699	0.586	0.833	< .0001	0.952	0.923	0.982	0.4878	0.758	0.650	0.884	0.0004
Clonazepam	0.903	0.624	1.308	0.5910	0.995	0.911	1.087	0.6012	0.924	0.658	1.297	0.6493
Gabapentin	0.904	0.716	1.140	0.3929	0.962	0.899	1.030	0.9345	0.913	0.729	1.144	0.4314
Lacosamide	0.743	0.456	1.211	0.2332	1.120	0.913	1.375	0.2374	0.803	0.504	1.280	0.3565
Lamotrigine	0.881	0.717	1.082	0.2256	0.964	0.910	1.021	0.2720	0.866	0.710	1.057	0.1573
Levetiracetam	1.033	0.874	1.220	0.7072	1.005	0.957	1.056	0.0207	1.063	0.907	1.246	0.4535
Oxcarbazepine	0.807	0.574	1.134	0.2168	1.017	0.928	1.114	0.0813	0.833	0.605	1.147	0.2625
Phenobarbital	0.833	0.455	1.523	0.5525	0.969	0.835	1.124	0.5023	0.835	0.463	1.504	0.5477
Phenytoin	0.684	0.516	0.908	0.0086	0.988	0.943	1.034	0.7551	0.816	0.641	1.039	0.0992
Pregabalin	0.979	0.749	1.278	0.8740	0.940	0.862	1.024	0.3630	0.955	0.739	1.235	0.7265
Primidone	1.042	0.743	1.461	0.8125	0.949	0.889	1.014	0.2533	1025	0.759	1.384	0.8705
Topiramate	0.810	0.535	1.226	0.3184	1.046	0.925	1.183	0.1650	0.868	0.582	1.296	0.4885
Valproate	0.937	0.779	1.126	0.4865	0.993	0.958	1.030	0.1958	0.964	0.815	1.140	0.6689

*ASM* antiseizure medication, *OR* odds ratio, *CI* confidence interval

Of note, CBZ was associated with a significant decreased incidence of cancer in Model I (ever used versus never use; OR 0.699, 95% CI 0.586–0.833, *p* < 0.0001) and Model III (effect per 5 years of therapy, OR 0.758, 95% CI 0.650–0.884, *p* < 0.0004). In the cancer subgroups, this effect was found most pronounced for digestive organs and skin (Table S1).

## Discussion

In this cohort study based on a German wide register from general practitioners, we looked at the possible associations of ASM use with cancer risk among PWE. The analyses followed the definition according to Shelby et al. that a relevant drug–cancer association should be significant, persistent over time, and carry a large relative risk [24]. Our main finding was that ASM administration did not significantly increase cancer incidence. This goes along with the results of several recently published epidemiological studies regarding this subject [4, 8, 13, 15, 25, 26].

Comorbidity in epilepsy patients is widespread. If cancer incidence is truly increased in PWE, various aspects of the epilepsies, such as diagnostic procedures or lifestyle factors, could be causal beside ASM use [3, 4]. To rule out confounding bias by epilepsy itself, people with epilepsy and ASM intake served as controls in the present study. In addition, we matched the groups according to age, sex, treating physician, index year and CCI to avoid further confounding patient or treatment characteristics. The CCI is a valid tool to measure the burden of prognostic comorbidities and is commonly used for risk adjustment in data analysis used in this study [23, 27]. The index does not explicitly include behavioral factors like smoking and alcohol use, eating habits or exposure to chemical noxae. However, these factors are risk factors for the comorbidities considered in the CCI.

We examined three models for each group and drug, one for any use effects (Model I, ever vs. never use) and two for long-term effects (Model II + III, effect per 1 or 5 therapy years). Carcinogenicity may be the result of genotoxic or non-genotoxic effects. For genotoxic effects, there is no safe exposure threshold or dose exists, i.e. short-term exposure may be sufficient to induce cancer. In contrast, non-genotoxic carcinogens have a safe exposure threshold and exert their carcinogenetic properties mostly in long-term use [28]. In our study, no ASM administration showed a positive drug–cancer association, neither for short-term nor long-term use. We detected a significant decrease in cancer risk under CBZ-therapy, namely for cancers of the skin and digestive system. Preclinical studies offer various models that would potentially explain cancer-suppressive effects of CBZ. For example, CBZ might prevent DNA damage induced by ionizing radiation, thus leading to a lower cancer risk (Kim et al., 2012). Additionally, CBZ may exert histone deacetylase (HDAC)-inhibitory activities [29]. HDACs are involved in modulating chromatin structure and gene expression; inhibition may increase tumor cell killing via complex mechanisms [30]. Still, our results should be carefully interpreted because the current study represents a retrospective association study which cannot prove causal relationships due to the design of the study. Another problem when dealing with this kind of retrospective data is the so-called protopathic bias which refers to the phenomenon that a drug may be started due to the first symptoms of a disease before its firm diagnosis and therefore, erroneously be suspected to cause the disease [31]. This error leads ultimately to overestimation of carcinogenetic effects of a drug. To avoid this, a time lag between exposure and cancer diagnoses may be included. Protopathic bias can be minimized by assessing various models over longer periods (effect per 1 and per 5 treatment years) which was applied to the present study. Regarding CBZ, our results may have been biased by similar effects. If diagnostics prompted by a first seizure show results suspicious for cancer, the patient might be rather put on a drug with low risk for interactions with oncological treatment strategies like LEV rather than on CBZ which has a high potential for interactions with various chemotherapies [32]. In fact, in our study, PWE with cancer were less likely to receive CBZ than PWE without cancer and LEV was more often used in PWE with cancer (see Table 1). Our retrospective design does, therefore, not allow to assume causal implications between CBZ and cancer suppression but might be useful generating new hypotheses for further research.

## Limitations

We only included patients who were treated by GPs, who care for PWE in Germany in addition to neurologists [33]. Nevertheless, we cannot safely assume that we covered the entire spectrum of patients. Since we relied on ICD-codes, we also cannot exclude under- or misdiagnoses of epilepsy by GP doctors although ICD coding by physicians has been shown to be reliable for identifying PWE [34]. We excluded neurologists because we could not assume that neurologists accurately code cancer diagnoses in addition to the epilepsy diagnosis. Moreover, cancer patients may have been treated exclusively by specialists, e.g. gynecologists in the case of breast cancer, leading to a lack of GP documentation regarding this type of cancer. Unfortunately, it was not possible to match patients according to the severity of their epilepsy syndrome because our data are based on retrospective analyses of registers which did not provide data on seizure load. We did not test for dose-dependent effects because it would have made the groups too small for reasonable statistical analyses. This is certainly a weakness of our study and warrants further research. We were not able to record and match the patient groups for tumor stadium according to the TNM-classification either. This may have disguised effects of ASM on tumor severity.

## Conclusion

In this retrospective analysis, we did not find any evidence that any of the investigated ASM increases the incidence of cancer in epilepsy patients. The decreased cancer risk under treatment with CBZ is of note but requires further prospective studies because bias cannot be excluded.

## Supplementary Information

Below is the link to the electronic supplementary material.

Electronic supplementary material 1 (DOCX 23 kb)

## Data Availability

The datasets generated during and/or analysed during the current study are available from the corresponding author on reasonable request.

## Abbreviations

ASM: Anti-seizure Medication
ATC: Anatomical Therapeutic Chemical
CBZ: Carbamazepin
CCI: Charlson Comorbidity Index
GP: General Practitioner
HDAC: Histone deacetylase
LEV: Levetiracetam
OR: Odds Ratio
PB: Phenobarbital
PHT: Phenytoin
PWE: People with Epilepsy
VPA: Valproate
