# Supplementary material for: Anti-seizure medication is not associated with an increased risk to develop cancer in epilepsy patients
Source: J Neurol. 2021 Jan 23;268(6):2185–91. doi: 10.1007/s00415-020-10379-4 (PMC8179889; doi:10.1007/s00415-020-10379-4)
Supplement: Supplementary file 1 — Electronic supplementary material 1 (DOCX 23 kb) [file 415_2020_10379_MOESM1_ESM.docx]

**Supplementary Table 1** Association between antiseizure medication and the incidence of cancer in epilepsy patients in German general practices by cancer entity

| ASM | Model I: ever use versus never use | | | |  | Model II: Effect per year of therapy | | | |  | Model III: Effect per 5 therapy years | | | |
| --- | --- | --- | --- | --- | --- | --- | --- | --- | --- | --- | --- | --- | --- | --- |
|  | OR | 95% CI | | P value |  | OR | 95% CI | | P value |  | OR | 95% CI | | P value |
| **Digestive organs** |  |  |  |  |  |  |  |  |  |  |  |  |  |  |
| CARBAMAZEPINE | 0.622 | 0.403 | 0.959 | 0.0317 |  | 0.946 | 0.870 | 1.028 | 0.1899 |  | 0.644 | 0.433 | 0.959 | 0.0303 |
| CLONAZEPAM | 0.846 | 0.351 | 2.037 | 0.7088 |  | 0.991 | 0.747 | 1.313 | 0.9479 |  | 0.888 | 0.370 | 2.133 | 0.7907 |
| GABAPENTIN | 0.652 | 0.373 | 1.141 | 0.1345 |  | 0.999 | 0.864 | 1.155 | 0.9890 |  | 0.731 | 0.430 | 1.243 | 0.2477 |
| LACOSAMIDE | 1.039 | 0.388 | 2.779 | 0.9395 |  | 1.149 | 0.755 | 1.748 | 0.5166 |  | 1.025 | 0.385 | 2.729 | 0.9612 |
| LAMOTRIGINE | 0.953 | 0.584 | 1.556 | 0.8475 |  | 0.983 | 0.864 | 1.119 | 0.7965 |  | 0.912 | 0.569 | 1.461 | 0.7016 |
| LEVETIRACETAM | 0.713 | 0.478 | 1.064 | 0.0974 |  | 0.957 | 0.866 | 1.058 | 0.3913 |  | 0.742 | 0.512 | 1.075 | 0.1142 |
| OXCARBAZEPINE | 1.107 | 0.461 | 2.658 | 0.8205 |  | 1.054 | 0.872 | 1.274 | 0.5841 |  | 1.177 | 0.542 | 2.559 | 0.6803 |
| PHENOBARBITAL | 1.458 | 0.407 | 5.227 | 0.5626 |  | 0.985 | 0.542 | 1.790 | 0.9608 |  | 1481 | 0.415 | 5.283 | 0.5446 |
| PHENYTOIN | 0.991 | 0.511 | 1.921 | 0.9786 |  | 1.032 | 0.940 | 1.135 | 0.5063 |  | 1.067 | 0.616 | 1.848 | 0.8173 |
| PREGABALIN | 0.954 | 0.490 | 1.859 | 0.8904 |  | 0.925 | 0.718 | 1.192 | 0.5466 |  | 0.945 | 0.486 | 1.839 | 0.8687 |
| PRIMIDONE | 1.909 | 0.820 | 4.445 | 0.1340 |  | 0.911 | 0.727 | 1.142 | 0.4207 |  | 1665 | 0.752 | 3.684 | 0.2086 |
| TOPIRAMATE | 0.927 | 0.288 | 2.984 | 0.8987 |  | 1.556 | 0.559 | 4.333 | 0.3972 |  | 0.968 | 0.300 | 3.120 | 0.9568 |
| VALPROATE | 0.755 | 0.480 | 1.188 | 0.2250 |  | 0.952 | 0.849 | 1.068 | 0.4041 |  | 0.821 | 0.537 | 1.256 | 0.3630 |
|  |  |  |  |  |  |  |  |  |  |  |  |  |  |  |
| **Respiratory organs** |  |  |  |  |  |  |  |  |  |  |  |  |  |  |
| CARBAMAZEPINE | 0.784 | 0.442 | 1.392 | 0.4062 |  | 0.966 | 0.849 | 1.100 | 0.6041 |  | 0.873 | 0.510 | 1.495 | 0.6206 |
| CLONAZEPAM | 0.836 | 0.275 | 2.538 | 0.7515 |  | 0.993 | 0.615 | 1.605 | 0.9774 |  | 0.880 | 0.286 | 2.708 | 0.8237 |
| GABAPENTIN | 1.212 | 0.563 | 2.609 | 0.6238 |  | 1118 | 0.881 | 1.418 | 0.3597 |  | 1278 | 0.592 | 2.758 | 0.5314 |
| LACOSAMIDE | 1.052 | 0.181 | 6.107 | 0.9552 |  |  |  |  |  |  | 1.302 | 0.313 | 5.415 | 0.7171 |
| LAMOTRIGINE | 1.219 | 0.614 | 2.421 | 0.5715 |  | 0.903 | 0.712 | 1.146 | 0.4003 |  | 1.248 | 0.631 | 2.468 | 0.5236 |
| LEVETIRACETAM | 0.993 | 0.558 | 1.770 | 0.9821 |  | 1.081 | 0.905 | 1.291 | 0.3917 |  | 1.089 | 0.625 | 1.898 | 0.7636 |
| OXCARBAZEPINE | 0.262 | 0.067 | 1.028 | 0.0548 |  | 0.480 | 0.201 | 1.144 | 0.0975 |  | 0.264 | 0.068 | 1.027 | 0.0547 |
| PHENOBARBITAL | 0.397 | 0.038 | 4.192 | 0.4426 |  | 1190 | 0.672 | 2.106 | 0.5505 |  | 0.414 | 0.039 | 4.406 | 0.4649 |
| PHENYTOIN | 0.555 | 0.199 | 1.545 | 0.2595 |  | 0.970 | 0.825 | 1.141 | 0.7135 |  | 0.780 | 0.335 | 1.815 | 0.5636 |
| PREGABALIN | 1.167 | 0.473 | 2.881 | 0.7370 |  | 0.994 | 0.819 | 1.207 | 0.9527 |  | 1141 | 0.514 | 2.534 | 0.7464 |
| PRIMIDONE | 0.915 | 0.308 | 2.717 | 0.8734 |  | 0.729 | 0.447 | 1.188 | 0.2042 |  | 0.793 | 0.319 | 1.969 | 0.6173 |
| TOPIRAMATE | 3.057 | 0.774 | 12.079 | 0.1109 |  | 2.498 | 0.766 | 8.144 | 0.1290 |  | 3.027 | 0.773 | 11.862 | 0.1119 |
| VALPROATE | 0.829 | 0.446 | 1.539 | 0.5522 |  | 0.983 | 0.873 | 1.107 | 0.7754 |  | 0.881 | 0.507 | 1.531 | 0.6533 |
|  |  |  |  |  |  |  |  |  |  |  |  |  |  |  |
| **Skin** |  |  |  |  |  |  |  |  |  |  |  |  |  |  |
| CARBAMAZEPINE | 0.538 | 0.333 | 0.868 | 0.0110 |  | 0.958 | 0.886 | 1.037 | 0.2873 |  | 0.692 | 0.456 | 1.051 | 0.0841 |
| CLONAZEPAM | 2.582 | 0.887 | 7.518 | 0.0819 |  | 1.313 | 0.855 | 2.016 | 0.2129 |  | 2.544 | 0.914 | 7.084 | 0.0738 |
| GABAPENTIN | 0.533 | 0.282 | 1.007 | 0.0525 |  | 0.886 | 0.717 | 1.095 | 0.2638 |  | 0.595 | 0.318 | 1.113 | 0.1039 |
| LACOSAMIDE | 2.275 | 0.395 | 13.092 | 0.3575 |  | 1.202 | 0.623 | 2.318 | 0.5835 |  | 2.228 | 0.389 | 12.754 | 0.3683 |
| LAMOTRIGINE | 0.951 | 0.549 | 1.648 | 0.8574 |  | 1.064 | 0.895 | 1.265 | 0.4815 |  | 1.008 | 0.584 | 1.740 | 0.9780 |
| LEVETIRACETAM | 0.970 | 0.606 | 1.552 | 0.8978 |  | 1.047 | 0.920 | 1.192 | 0.4887 |  | 1.101 | 0.708 | 1.713 | 0.6679 |
| OXCARBAZEPINE | 0.742 | 0.285 | 1.931 | 0.5405 |  | 0.964 | 0.796 | 1.167 | 0.7074 |  | 0.857 | 0.376 | 1.955 | 0.7145 |
| PHENOBARBITAL | 1.164 | 0.248 | 5.466 | 0.8475 |  | 1.078 | 0.772 | 1.505 | 0.6584 |  | 1.247 | 0.268 | 5.810 | 0.7786 |
| PHENYTOIN | 0.602 | 0.289 | 1.254 | 0.1752 |  | 0.920 | 0.806 | 1.051 | 0.2204 |  | 0.740 | 0.389 | 1.408 | 0.3590 |
| PREGABALIN | 0.818 | 0.408 | 1.642 | 0.5720 |  | 0.881 | 0.710 | 1.093 | 0.2502 |  | 0.779 | 0.408 | 1.486 | 0.4486 |
| PRIMIDONE | 1.100 | 0.439 | 2.753 | 0.8394 |  | 1.194 | 0.904 | 1.577 | 0.2123 |  | 1.278 | 0.548 | 2.984 | 0.5703 |
| TOPIRAMATE | 0.190 | 0.038 | 0.946 | 0.0426 |  | 0.544 | 0.193 | 1.533 | 0.2498 |  | 0.207 | 0.042 | 1.032 | 0.0547 |
| VALPROATE | 1.077 | 0.660 | 1.758 | 0.7671 |  | 1.036 | 0.946 | 1.136 | 0.4434 |  | 1.186 | 0.758 | 1.855 | 0.4543 |
|  |  |  |  |  |  |  |  |  |  |  |  |  |  |  |
| **Breast** |  |  |  |  |  |  |  |  |  |  |  |  |  |  |
| CARBAMAZEPINE | 0.865 | 0.451 | 1.657 | 0.6618 |  | 1.018 | 0.917 | 1.131 | 0.7349 |  | 0.934 | 0.539 | 1.620 | 0.8080 |
| CLONAZEPAM | 0.811 | 0.208 | 3.160 | 0.7632 |  | 0.946 | 0.673 | 1.328 | 0.7475 |  | 0.828 | 0.214 | 3.208 | 0.7851 |
| GABAPENTIN | 1.334 | 0.606 | 2.937 | 0.4743 |  | 0.830 | 0.638 | 1.078 | 0.1626 |  | 1329 | 0.606 | 2.916 | 0.4772 |
| LACOSAMIDE | 1.505 | 0.176 | 12.879 | 0.7092 |  | 2.033 | 0.372 | 11.118 | 0.4129 |  | 1.516 | 0.180 | 12.782 | 0.7021 |
| LAMOTRIGINE | 0.507 | 0.239 | 1.077 | 0.0773 |  | 0.703 | 0.492 | 1.003 | 0.0522 |  | 0.483 | 0.236 | 0.989 | 0.0465 |
| LEVETIRACETAM | 1.058 | 0.558 | 2.006 | 0.8630 |  | 1.160 | 0.933 | 1.444 | 0.1813 |  | 1.030 | 0.551 | 1.924 | 0.9270 |
| OXCARBAZEPINE | 0.470 | 0.128 | 1.719 | 0.2537 |  | 0.948 | 0.700 | 1.283 | 0.7294 |  | 0.517 | 0.159 | 1.689 | 0.2749 |
| PHENOBARBITAL |  |  |  |  |  |  |  |  |  |  |  |  |  |  |
| PHENYTOIN | 0.547 | 0.190 | 1.574 | 0.2630 |  | 0.957 | 0.842 | 1.088 | 0.5039 |  | 0.799 | 0.396 | 1.613 | 0.5319 |
| PREGABALIN | 1.188 | 0.465 | 3.033 | 0.7189 |  | 0.934 | 0.727 | 1.200 | 0.5938 |  | 1103 | 0.463 | 2.627 | 0.8241 |
| PRIMIDONE | 2.123 | 0.647 | 6.965 | 0.2143 |  | 0.969 | 0.869 | 1.081 | 0.5705 |  | 1310 | 0.522 | 3.288 | 0.5646 |
| TOPIRAMATE | 0.657 | 0.099 | 4.344 | 0.6629 |  | 1.229 | 0.794 | 1.903 | 0.3555 |  | 0.667 | 0.102 | 4.379 | 0.6732 |
| VALPROATE | 1.167 | 0.557 | 2.441 | 0.6826 |  | 1.078 | 0.911 | 1.275 | 0.3846 |  | 1.169 | 0.580 | 2.355 | 0.6624 |
|  |  |  |  |  |  |  |  |  |  |  |  |  |  |  |
| **Prostate** |  |  |  |  |  |  |  |  |  |  |  |  |  |  |
| CARBAMAZEPINE | 1.013 | 0.494 | 2.078 | 0.9717 |  | 1.046 | 0.940 | 1.164 | 0.4068 |  | 1.272 | 0.713 | 2.271 | 0.4157 |
| CLONAZEPAM |  |  |  |  |  |  |  |  |  |  |  |  |  |  |
| GABAPENTIN | 1.173 | 0.486 | 2.830 | 0.7222 |  | 1.177 | 0.872 | 1.587 | 0.2869 |  | 1.281 | 0.535 | 3.068 | 0.5779 |
| LACOSAMIDE |  |  |  |  |  |  |  |  |  |  |  |  |  |  |
| LAMOTRIGINE | 1.310 | 0.522 | 3.291 | 0.5655 |  | 1.125 | 0.874 | 1.449 | 0.3591 |  | 1.518 | 0.609 | 3.782 | 0.3706 |
| LEVETIRACETAM | 0.679 | 0.344 | 1.340 | 0.2646 |  | 0.984 | 0.828 | 1.171 | 0.8596 |  | 0.848 | 0.452 | 1.590 | 0.6067 |
| OXCARBAZEPINE | 1.115 | 0.290 | 4.284 | 0.8743 |  | 1.984 | 0.586 | 6.722 | 0.2712 |  | 1.265 | 0.330 | 4.859 | 0.7317 |
| PHENOBARBITAL |  |  |  |  |  |  |  |  |  |  |  |  |  |  |
| PHENYTOIN | 0.254 | 0.074 | 0.880 | 0.0306 |  | 0.946 | 0.828 | 1.081 | 0.4161 |  | 0.510 | 0.205 | 1.269 | 0.1478 |
| PREGABALIN | 1.783 | 0.507 | 6.273 | 0.3679 |  | 1.812 | 0.818 | 4.014 | 0.1429 |  | 1.966 | 0.561 | 6.889 | 0.2908 |
| PRIMIDONE | 1.611 | 0.350 | 7.416 | 0.5401 |  | 0.925 | 0.739 | 1.159 | 0.4993 |  | 1193 | 0.381 | 3.739 | 0.7622 |
| TOPIRAMATE | 1.082 | 0.228 | 5.131 | 0.9208 |  | 1.657 | 0.402 | 6.839 | 0.4847 |  | 1.287 | 0.330 | 5.026 | 0.7163 |
| VALPROATE | 0.929 | 0.422 | 2.044 | 0.8550 |  | 1.013 | 0.887 | 1.157 | 0.8479 |  | 1.081 | 0.533 | 2.193 | 0.8299 |

ASM = antiseizure medication, OR = odds ratio, CI = confidence interval
